# Dataset for predicting single-spot proton ranges in proton therapy of prostate cancer

**DOI:** 10.1038/s41597-021-01028-0

**Published:** 2021-09-29

**Authors:** Hugo Freitas, Paulo Magalhaes Martins, Thomas Tessonnier, Benjamin Ackermann, Stephan Brons, Joao Seco

**Affiliations:** 1 German Cancer Research Center – DKFZ, Heidelberg, Germany; 2 Departamento de Física e Astronomia, Faculdade de Ciências da Universidade do Porto, Porto, Portugal; 3 Instituto de Biofísica e Engenharia Biomédica, Faculdade de Ciências da Universidade de Lisboa, Lisboa, Portugal; 4 Heidelberg Ion-Beam Therapy Center (HIT), Department of Radiation Oncology, Heidelberg University Hospital, Heidelberg, Germany; 5 Department of Physics and Astronomy, University of Heidelberg, Heidelberg, Germany

**Keywords:** Techniques and instrumentation, Prostate cancer, Biomedical engineering, Radiotherapy

## Abstract

The number of radiotherapy patients treated with protons has increased from less than 60,000 in 2007 to more than 220,000 in 2019. However, the considerable uncertainty in the positioning of the Bragg peak deeper in the patient raised new challenges in the proton therapy of prostate cancer (PCPT). Here, we describe and share a dataset where 43 single-spot anterior beams with defined proton energies were delivered to a prostate phantom with an inserted endorectal balloon (ERB) filled either with water only or with a silicon-water mixture. The nuclear reactions between the protons and the silicon yield a distinct prompt gamma energy line of 1.78 MeV. Such energy peak could be identified by means of prompt gamma spectroscopy (PGS) for the protons hitting the ERB with a three-sigma threshold. The application of a background-suppression technique showed an increased rejection capability for protons hitting the prostate and the ERB with water only. We describe each dataset, document the full processing chain, and provide the scripts for the statistical analysis.

## Abstract (summary)

**Table:** 

Measurement(s)	single-spot proton ranges • surface of prostate
Technology Type(s)	prompt gamma spectroscopy
Factor Type(s)	campaign • beam energy • processing

Machine-accessible metadata file describing the reported data: 10.6084/m9.figshare.15156039

## Background & Summary

The use of endorectal balloons (ERBs) for stabilizing the prostate movement during radiotherapy has been applied to three dimensional conformal radiotherapy (3D CRT)^1–3^ and intensity-modulated radiotherapy (IMRT)^4–7^. A dose reduction to the rectal wall by means of an ERB has been observed by several authors^1,8,9^. Such medical device could be applied to proton therapy of prostate cancer (PCPT) also to avoid organ movement and to serve as a range probe for monitoring the Bragg peak position before reaching the rectal wall.

Several clinical studies indicated increased toxicity for PCPT, when compared to standard conventional photon treatments^10,11^. However, the most comprehensive studies were carried out when proton therapy was in its relative infancy and only passively-scattered proton therapy (PSPT) was available. More recent studies have demonstrated more favorable toxicity outcomes with proton therapy^12–14^.

Prompt gamma spectroscopy emerged as one of the most promising techniques to monitor real-time the proton range with millimetric precision^15^. Such technique relies on the measurement of the prompt gamma energy spectra following the nuclear reactions of the protons with the human tissue. Elements present in the human body, such as oxygen and carbon were assessed in terms of their contribution to the proton^16^, helium^17^, and carbon range^18^. Other elements usually not present in the human body (e.g., Aluminum and Titanium) were also studied by means of PGS with a new kind of scintillator detectors–CeBr_3_^17,19^. Such detectors also allowed measuring concentrations of such elements in different tissue surrogates^20^.

In the related work published in Scientific Reports^21^ the same authors demonstrated the feasibility of an ERB filled with a mixture of water and silicon to serve as a range probe during a standard 2 Gy PCPT treatment fraction. Here, we present a PGS dataset that was obtained after 43 single-spot irradiations of the same prostate phantom with the inserted range probe by anterior proton beams. The data obtained from two measurement campaigns provided enough evidence to determine the presence of the silicon in the beam path above a certain beam energy. Such evidence is crucial to monitor the irradiation of the rectal wall in anterior beams and may open new possibilities for future control or prevention. The delivery of very low dose scout beams prior to the treatment in order to evaluate whether the beam stops in the prostate or in the ERB has already been proposed by Hoesl *et al*.^22^.

With this work, we believe such technique may be verified in other proton centers around the world with strong potential to be soon translated to the clinical practice. All the materials and methods presented may be easily available to other researchers. The energies used in this work are also within the range of energies available in most proton centers either with passive scattering or active scanning delivery. It is also our purpose to stimulate others to reuse the present data for developing new fitting models and statistical tools as well as considering other phantoms, range probes and geometries. Finally, data from such comprehensive studies could be used to further expand the application of proton therapy to other targets which are not currently considered due to the close proximity to other organs at risk.

## Methods

### Prostate phantom

The phantom is a prostate training phantom, CIRS^Ⓡ^ model 070 L (CIRS Inc., Norfolk, USA). It is commonly used for ultrasound images and to be biopsied through the Z-Skin^TM^ rectal wall or perineal membrane. The main inner composition is Zerdine^Ⓡ^. It still includes a urethra with a diameter of 0.7 cm, seminal vesicles with a diameter of 0.7 cm and 10 cm long, and two lesions. The container has a volume of 9 cm × 10 cm × 10 cm and a probe opening of 1.2 cm.

### Endorectal balloon

The ERB is a QLRAD^Ⓡ^ Rectal Pro75^TM^ (QLRAD International, Larnaca, Cyprus) commonly used to stabilize the prostate movement in radiotherapy. It is coupled to a syringe via a smaller tube and a latch closes the liquid flow. The ERB was filled with 50 mL.

### Water silicon mixture

The mixture of water and silicon dioxide (SiO_2_) consisted of 90 mL of deionized water and 60 g of diatomaceous earth (*Kieselgur*) from Health Leeds^Ⓡ^ (Health Leeds UK Ltd, Horeb, UK).

### The HIT facility

The Heidelberg Ion-Beam Therapy Center - HIT^23^ accelerates proton, helium, carbon, and oxygen ions from 48 MeV/u up to 430 MeV/u. While protons and carbon ions are routinely implemented in the clinical setting, helium ions are currently being commissioned^24,25^, and oxygen ions still remain as a research beam species.

The intensities in clinical practice range from 2 × 10^6^ p/s for carbon ions to 3.2 × 10^9^ p/s for protons. There are two horizontal rooms and a 360° gantry for therapy. There is a horizontal experimental room where all the experiments referred in this paper were performed.

### Computed tomography

The computed tomography (CT) followed the routine CT protocol for ion beam therapy planning at HIT with the Siemens SOMATOM Confidence^Ⓡ^ RT Pro (Siemens Healthineers, Erlangen, Germany). The phantom and the inserted ERB were scanned with a tube voltage of 120 kV and the image was reconstructed for a field of view (FOV) of 50 cm with a convolution kernel B40s and a spacing between slices of 3 mm.

**Fig. 1:**
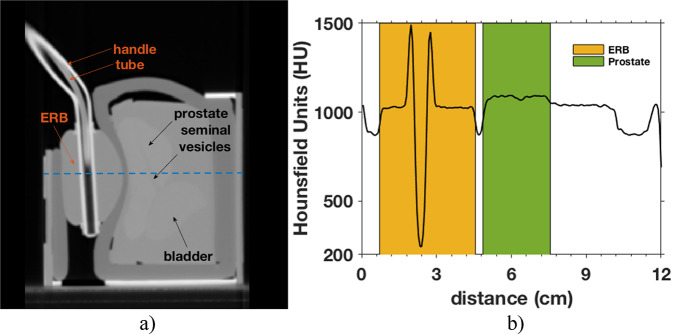
Left: Image of a CT of the prostate training phantom CIRS Model 070 L (sagittal view). Right: The profile in Hounsfield Units (HU) was taken over the blue dashed line.

In Fig. 1, we show a CT of the prostate phantom and the ERB. Both ImageJ^Ⓡ^ and MATLAB^Ⓡ^ software were used to measure the ERB diameter (*ϕ*_*ERB*_ ≈ 4 cm) and the gap between the ERB and the prostate (gap ≈ 0.3–0.5 cm). The measures were taken manually at 50% of the slope transition between structures. The gap values between the rectum wall and the prostate are slightly higher than the mean values reported in the literature^26,27^.

### Experimental setup

**Fig. 2:**
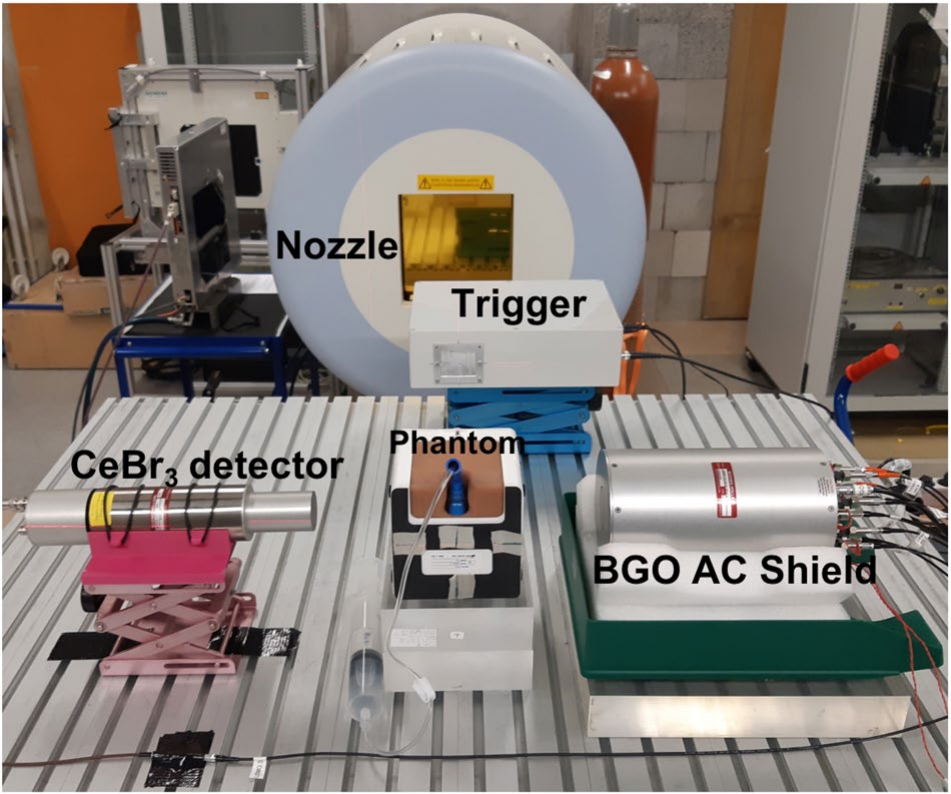
Experimental setup used to obtain the prompt gamma spectra following the nuclear reactions of anterior proton beams with a patient-like prostate phantom. The beam trigger and the BGO AC shield were used for background suppression.

The main components of the experimental setup are the nozzle, the target, the CeBr_3_ detectors, the trigger, and the BGO anti-coincidence (AC) Compton suppressor (see Fig. 2).

The CeBr_3_ detectors are scintillation detectors with very good time and energy resolution. They feature a measured energy resolution of 3.49%^17^ and a measured time resolution in coincidence with the trigger of 0.85 ns FWHM^28^. They are mainly used for range verification of the proton and ion beams in the patient. The CeBr_3_ detectors were aligned with the isocenter and positioned at a distance of 15 cm from the beam axis. This distance follows from previous studies^20^ as a standard positioning for such detectors and represents a tradeoff between the count rate and the limits to the system throughput. The CeBr_3_ crystals are identical in size (diameter d = 3.81 cm and length l = 7.62 cm). One crystal was coupled to a Hamamatsu R13089 photomultiplier tube (PMT) and the other one to a Hamamatsu R9420-100 PMT. The former one was only used in the validation campaign. Both detectors were plugged to a voltage divider.

The secondary detector is constituted by BGO crystals sectioned in eight optically separated and azimuthally symmetric segments. Each section is optically coupled to an independent Hamamatsu PMT R1924 followed by a pre-amplified circuit. The eight individual components are contained in a cylindrical shape and hollow cylinder to fit one of the CeBr_3_ detectors. The BGO with energy- and time-resolved signal was used as an AC shield allowing the background suppression of Compton and single and double escape events. The time resolution between the CeBr_3_ and AC is 3.58 ns FWHM^17^. The BGO detector was only used in the main campaign.

In order to enable time-of-flight measurements, the prototype is equipped with an array of scintillating fibers (beam trigger) with a decay time of 3.2 ns and a sub-nanosecond intrinsic time resolution of 0.7 ns FWHM^28^. The fibers (BCF-12 from Saint Gobain Crystals) have a diameter of 0.5 mm and are coupled in an alternate fashion to two Hamamatsu R657 PMTs. The fibers and PMTs are enclosed in a light-shielding box with an external window for the beam. The beam trigger provides time information to derive the TOF spectrum allowing the background suppression of uncorrelated events (e.g., hydrogen neutron capture).

The anode output of each detector fed the data acquisition system (DAQ)^29^. This is a module of a FlashCam FADC system, originally designed for the Cherenkov Telescope Array (CTA)^30^.

### Intensities, acquisition times, and counts

The results shown were obtained with an intensity of 8 × 10^7^ p/s and the acquisition lasted 1:07 min (14 spills). A total of 4.69 × 10^9^ protons were delivered. The counts ranged from 1.39 × 10^6^ for an energy of 86.72 MeV to 2.13 × 10^6^ for an energy of 128.11 MeV.

### Main, reference, and validation campaigns

A main campaign comprised 23 measurements ranging from 86.72 MeV to 134.06 MeV. Table 1 shows the relative range starting at the end of the phantom as well as the target regions being hit. Another campaign consisting of 10 measurements each acquired data for an ERB filled only with water (reference) and with the water silicon mixture (validation). Such measurements were performed for a region comprehending part of the prostate, the rectal wall (RW), and the ERB.

**Table 1:** Two campaigns comprising 43 measurements with different beam energies ranging from 86.7 MeV to 134.1 MeV.

#	Energy (MeV)	Campaign		*D*_*end*_ (cm)	Target
		Main	Reference & Validation		
1	86.72	✓		7.4	prostate
2	90.70	✓		6.9	prostate
3	94.54	✓		6.4	prostate
4	96.05	✓		6.2	prostate
5	97.53	✓		6.0	prostate
6	98.27		✓	5.9	prostate
7	99.01	✓		5.8	prostate
8	100.46	✓		5.6	prostate
9	101.18	✓	✓	5.4	RW
10	103.32	✓	✓	5.2	RW
11	104.03	✓		5.1	RW
12	104.73	✓	✓	5.0	RW
13	105.43	✓	✓	4.9	ERB
14	106.12	✓	✓	4.8	ERB
15	107.51	✓	✓	4.6	ERB
16	108.88	✓	✓	4.4	ERB
17	112.25	✓	✓	3.9	ERB
18	115.55	✓	✓	3.4	ERB
19	118.78	✓		2.9	handle
20	121.95	✓		2.4	handle
21	125.06	✓		1.9	ERB
22	128.11	✓		1.4	ERB
23	131.11	✓		0.9	RW
24	134.06	✓		0.4	RW

The column *D*_*end*_ refers to the distance to the end of the prostate phantom. It spanned from 0.4 cm to 7.4 cm for the main campaign and from 3.4 cm to 5.9 cm for the reference and validation campaign. The latter comprised neighbouring regions including parts of the prostate, the rectal wall (RW), and the ERB.

### Processing

**Fig. 3:**
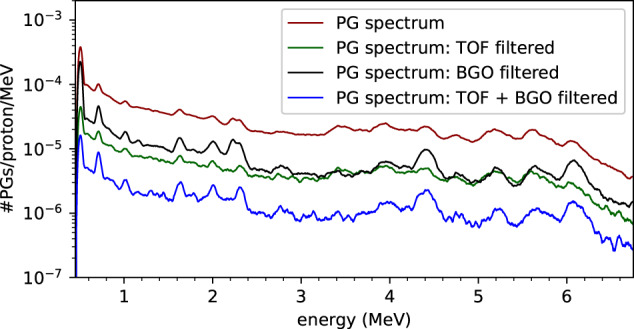
Prompt gamma (PG) spectra with and without background suppression methods.

In the two campaigns, the energy spectra were obtained after evaluating every event trace. An exponential modified Gaussian (EMG) was applied to a maximum of three peaks in each trace. Every fit has three parameters which were calculated numerically: area (*A*), mode (*m*), and height (*max*). Some events were discarded, such as overflows and pile-up. An *R-*squared distribution was determined and only the events above a certain threshold ($th{r}_{{r}_{adjusted}^{2}}=\mu -7.5\sigma$) were accepted. The knowledge of spill structure also allowed for the selection of the in-spill events^28^. The dead time was always below 15% and for this purpose a non-paralyzable dead-time correction was applied. A threshold for both low and high energy events was also applied as well as a smoothing with the *Savitzky–Golay* filter. For spectra calibration purposes, we calculated a calibration curve based on the spectral line *E*_*γ*_ = 0.6617 MeV following the ^137^*Cs* decay as well as on known oxygen energy spectral lines for fine tuning and correction for nonlinearity^17^. The calibration was further verified with spectral lines from other elements, such as ^28^Si. Finally, a calibrated energy spectrum was obtained as shown in Fig. 3.

The background suppression comprehends TOF and AC Compton suppression methods. The TOF measurements were provided by the arrival time of the protons to the scintillating fibers and the arrival time of the prompt gamma rays to the CeBr_3_ detectors. A time window of 10 ns was defined for the prompt gamma component. The signals on the BGO detector that arrived after the signals on the CeBr_3_ were recorded in anti-coincidence and an energy cut at 225 keV was applied. The several consecutive suppression steps are depicted in Fig. 3. The TOF + BGO filtered spectrum consists of only 6% of the total events depicted in the PG spectrum.

### Statistical analysis

The statistical analysis aimed at inferring whether the restricted model was sufficient to explain the data or an unrestricted model was otherwise needed. A region in the energy spectra from 1.58 MeV to 1.84 MeV was defined where either one peak at 1.635 MeV from oxygen de-excitation was present or instead an additional 1.78 MeV resulting from the de-excitation of the silicon was also present. A null hypothesis was defined to ascertain if the restricted model was sufficient to fit the data. The unrestricted model considered a Cauchy-Lorentz distribution for each de-excitation peak where the parameters are given by the amplitude, mean, sigma, and a constant value. On the other hand, the restricted model only considered a single Cauchy-Lorentz distribution. The observations depend on the binning and are in the order of 65 for the given energy interval. The degrees of freedom (*df*) are obtained from the difference between the number of observations and the number of estimated parameters. The *F statistic* or F-test allows determining if *H*_0_ is rejected and if the dropped independent variables are jointly statistically significant or insignificant at the appropriate significance level^31^. The *F statistic* is defined by $F\equiv \frac{(SS{R}_{r}-SS{R}_{ur})/q}{SS{R}_{ur}\,/(n-k-1)}$, where *SSR*_*r*_ is the sum of squared residuals from the restricted model and *SSR*_*ur*_ is the sum of squared residuals from the unrestricted model. The difference in degrees of freedom in the numerator is $q=d{f}_{r}-d{f}_{ur}=3$ and the denominator degrees of freedom are *df*_*ur*_ = *n* − *k* − 1 = 65 − 6 = 49. Once we have all the variables to compute the *F statistic*, we can assess for every measurement if the null hypothesis can be rejected with a three-sigma threshold, i.e., if can we reject *H*_0_ at 0.01% level.

## Data Records

**Fig. 4:**
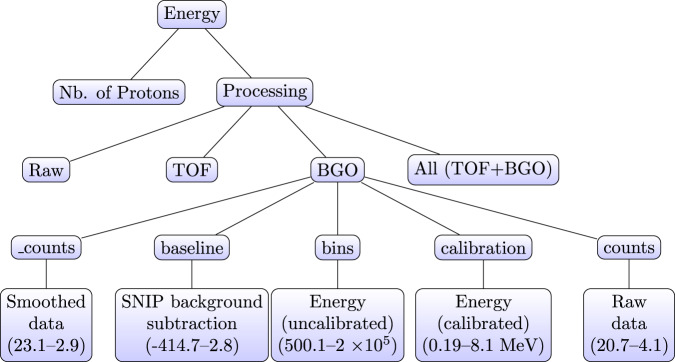
Data structure: For every proton energy, there is one layer with the number of protons irradiated and three further layers corresponding to background suppression steps (the raw data was not corrected for background). The TOF data considered a prompt-gamma window of 10 ns. The BGO data was obtained in anti-coincidence with the CeBr_3_ data and rejected events above 225 keV. All data represents the data obtained after the TOF and BGO cuts. Every layer contains five arrays with 2048 elements. Representative values for the ranging interval after the BGO cut are presented for a beam energy of 115.55 MeV.

The dataset is available at figshare^32^. The *maincampaign.pkl*, *referencecampaign.pkl*, and *validationcampaign.pkl* contain the energy spectra for the two campaigns (main campaign and reference & validation campaign). They have a similar three-layer nested structure (see Fig. 4), and were stored as python dictionaries using the pickle library. The top layer indicates the energy used in the experiments, while the second layer is subdivided in the follow dictionaries: *Raw*, *BGO*, *TOF*, and *All*. The *raw* data was not corrected for background, while the *TOF* data applied a time cut for a prompt-gamma window of 10 ns. Such cut removed 87% of the events. The *BGO* data was obtained in anti-coincidence with the CeBr_3_ data and rejected events above 225 keV. Such cut removed 68% of the events. *All* data represent the data obtained after the TOF and BGO cuts and the number of events left are approximately 6%. The number of protons for each of the 43 runs is given by the *proton* variable.

The third layer has five arrays: *_counts*, *baseline*, *bins*, *calibration*, and *counts*. The *_counts* array is given by the prompt gamma entries in the bin after applying a *Savitzky*–*Golay* smoothing filter. The *baseline* array was obtained after applying the non-linear iterative peak (SNIP) technique^33,34^ for background subtraction. The *bins* array contains the energy information after the time integration of the CeBr_3_ peaks in every trace (for a maximum of 3 peaks). The *calibration* array presents the energy information after applying a calibration curve based on known oxygen energy lines. The *counts* array is given by the prompt gamma entries in the bin without smoothing. Raw and smoothed spectra with 2048 bins (before or after calibration) can be obtained for every beam energy with and without background suppression techniques. Exemplary ranging intervals are presented in the last layer of Fig. 4 for the data obtained during the main campaign for a beam energy of 115.55 MeV and after BGO AC suppression.

## Technical Validation

**Fig. 5:**
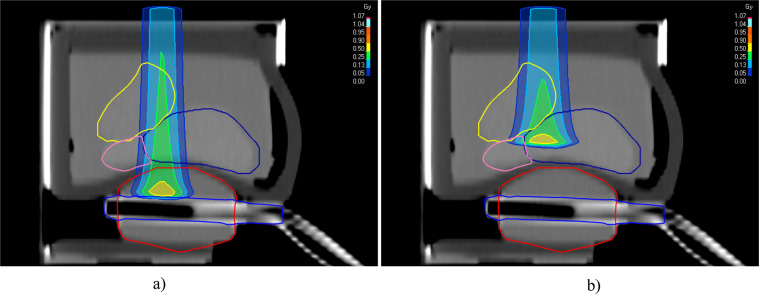
Dose distribution for a proton beam of 112.25 MeV (left) and 94.54 MeV (right). The structures in the prostate phantom are identified with blue (prostate), red (balloon) and yellow (bladder) contour lines.

In the two campaigns, we irradiated with single spots different regions of the phantom by increasing the energy of the beam in sequential steps. Figure 5 shows the dose deposition of a 112.25 MeV (left) and a 94.54 MeV (right) proton beams. The latter stops in the prostate while the former stops in the ERB.

**Fig. 6:**
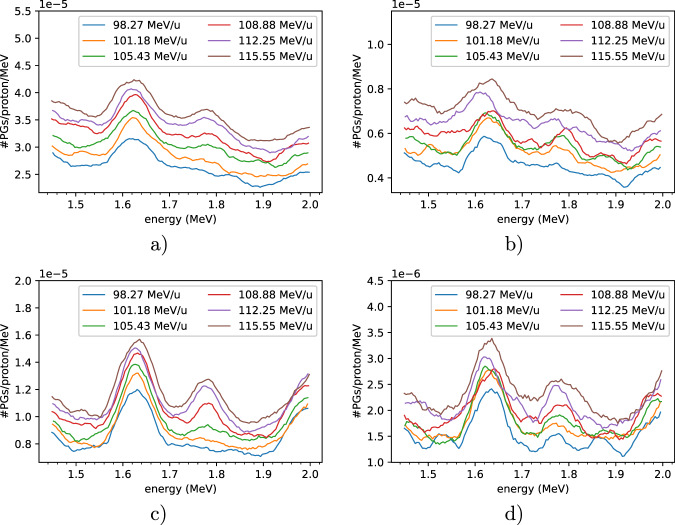
PG spectra for proton beams with energies ranging from 98.27 MeV up to 115.55 MeV. The spectra are zoomed in a region of interest including the 1.635 MeV peak from the oxygen de-excitation and the 1.78 MeV from the silicon de-excitation. Different background suppression techniques were used: (**a**) raw data; (**b**) after TOF cut; (**c**) after BGO AC suppression; (**d**) after TOF + BGO.

For every energy from both campaigns we obtained an energy spectrum. Figure 6 shows six energy spectra for six different energies. The background suppression techniques were consecutively applied thus rendering increased prominences for the 1.78 MeV peak after the BGO AC suppression. The TOF cuts have a strong impact in the available statistics thus making the quantification unreliable.

**Fig. 7:**
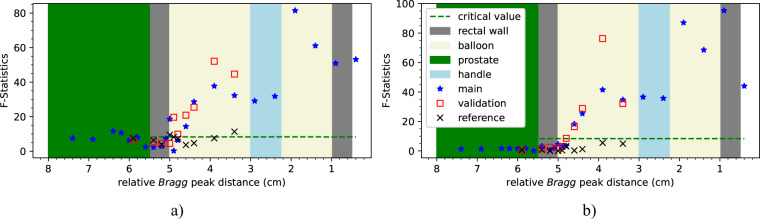
F-test for the measurements from both campaigns. The starred values were obtained in the main campaign and the squared and crossed values were obtained in the validation and reference measurements, respectively. Four irradiated regions are identified according to Table 1. The results after BGO AC suppression are presented on the right. The critical value was calculated for every measurement and only varies with the degrees of freedom.

We performed an F-test for every measurement from both campaigns. We tested if we could reject the restricted model with only 3 parameters from a single Cauchy-Lorentz distribution at a 0.01% level. The results for the different energies are presented in Fig. 7. A comparison with the results obtained after the BGO AC suppression are also presented (see Fig. 7, right). All measurements falling in the prostate and in the rectal wall are below the critical value after the BGO AC suppression as well as the reference measurements with the ERB filled with water. The measurements falling in the ERB filled with the water silicon mixture starting at the point 4.6 cm until the end of the phantom could reject the null hypothesis at 0.01% level, i.e., a single Cauchy-Lorentz distribution could not fit the peaks in the given energy interval. The absence of the water silicon mixture in the handle is clearly evident. The F-test presents most values for the main and validation experiments above the critical value for distances to the end of the phantom below 4.6 cm. The BGO AC suppression has a slightly better rejection capability. On the other hand, in the reference measurements and in the measurements without BGO AC suppression, type I errors are more likely to happen.

**Fig. 8:**
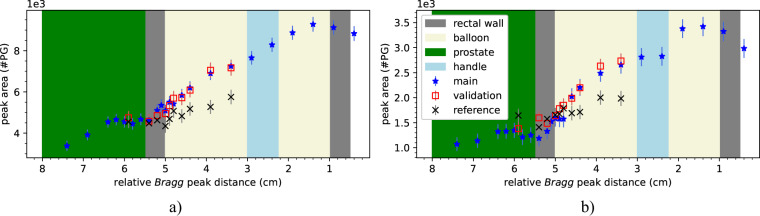
Relationship between the the 1.78 MeV peak area and the relative Bragg peak distance for measurements without background suppression (left) and with BGO AC suppression (right).

In order to quantify the 1.78 MeV silicon peak for different energies we determined the total area under the peak. The results with and without BGO AC suppression are shown in Fig. 8. The evolution trend for increasing energies shows great potential for predicting with single spots the proton range in PCPT, more specifically, within the ERB. The effect of the absence of the mixture in the handle is visible in the main campaign in both scenarios (with and without background suppression). The mean relative error between the main and validation campaign was 2% for the measurements without background suppression and 11% for the measurements with BGO AC suppression. Such results indicate a good reproducibility between campaigns.

**Fig. 9:**
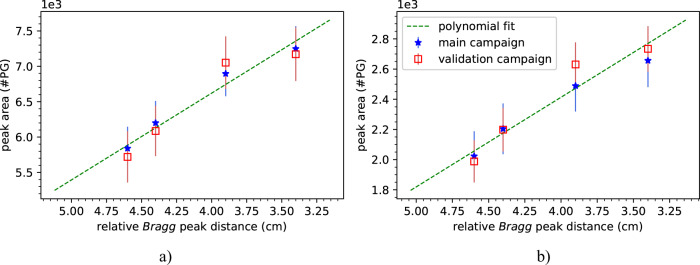
Linear fit to the main and validation campaign measurements presented in Fig. 8. The fit considered the region from 3.4 cm to 4.6 cm (distance from the inflated ERB surface to the handle).

Figure 9 shows the linear fit to the data presented in Fig. 8 for the interval from 3.4 cm to 4.6 cm. Such distance comprises the region from the inflated ERB surface to the handle and provides evidence of how far the proton beam went into the ERB. Moreover, we selected such a region since it corresponds to the energy levels in Table 1 where the main and validation campaigns match. Both models were compared with the expected results (see Table 2).

**Table 2:** Results of the linear fit model to measured data applied to the main and validation campaigns.

expected distances (cm)	distances without background suppression		distances with background suppression	
	*main campaign (cm)*	*validation campaign (cm)*	*main campaign (cm)*	*validation campaign (cm)*
4.60	4.64	4.73	4.66	4.72
4.40	4.34	4.44	4.35	4.36
3.90	3.78	3.65	3.87	3.63
3.40	3.49	3.55	3.59	3.46

The root mean square error (RMSE) was calculated for both cases (with BGO AC suppression and without background suppression). The former presented an RMSE of 1.03 mm and 1.49 mm while the latter presented an RMSE of 0.83 mm and 1.63 mm for the main and validation campaigns, respectively.

## Usage Notes

This dataset can be downloaded through the link mentioned above. Users of this dataset are expected to cite this paper in any research output generated from using this dataset as well as appropriately acknowledge the contributions of this dataset.

After downloading the datasets included in the *pickle* files, users can run the scripts to perform an F-test to every point in both campaigns. Users can also plot the energy spectra from each measurement including either the *raw* spectra or the ones after applying the background suppression techniques.

## Data Availability

All code is available in the figshare repository^32^. The code includes scripts for the analyses presented in this paper. The scripts rely on open source Python packages such as numpy^35^, pandas^36^, matplotlib^37^, scipy^38^, sklearn^39^ and pickle^40^.
